# Physiological and Biochemical Mechanisms of *Aoria nigripes* (Coleoptera, Chrysomelidae) Adaption to Flavonoid-Rich Plant *Nekemias grossedentata*

**DOI:** 10.3390/insects16040399

**Published:** 2025-04-10

**Authors:** Zhengwen Yu, Chenju Yang, Lian Xie, Feng Yang, Yuyu Yuan

**Affiliations:** School of Life Sciences, Guizhou Normal University, Guiyang 550025, China

**Keywords:** *Aoria nigripes*, *Nekemias grossedentata*, secondary metabolites, detoxifying enzymes, protective enzymes

## Abstract

This study aimed to explore the physiological and biochemical mechanisms of the interaction between *N. grossedentata* and *A. nigripes*. First, specimens were categorized into low- (6.16% ± 0.66%), medium- (9.23% ± 1.19%), and high-content groups (21.23% ± 1.23%) based on the initial dihydromyricetin concentration in *N. grossedentata*. Subsequently, we assessed the variations in total flavonoids, dihydromyricetin, myricitrin, and myricetin in plants 24, 48, and 72 h post-feeding. Concurrently, we analyzed the impact of plant leaf consumption on the detoxifying [glutathione S-transferase (GST), carboxylesterase (CarE), acetylcholinesterase (AchE), and cytochrome P450 (CYP450)] and protective enzyme [superoxide dismutase (SOD), peroxidase (POD) and catalase (CAT)] activities in *A. nigripes*, along with its metabolic processes. The results demonstrated that *N. grossedentata* enhanced its secondary metabolites, particularly dihydromyricetin, as a defensive response to insect-induced stress. *A. nigripes* utilized its detoxification and protective enzyme systems to mitigate the effects of high flavonoid levels in the host plant, with particular emphasis on the roles of detoxification enzymes (GST, AchE, CYP450, and CarE) in detoxification metabolism, which showed significant correlation (*p* < 0.01) with dihydromyricetin, exhibiting correlation coefficients of 0.689, 0.633, 0.579, and 0.561, respectively. Additionally, key flavonoids in *N. grossedentata* were observed to accumulate with different degrees during digestion and metabolism in insects. These findings lay a theoretical foundation for the further exploration of the molecular mechanisms of *A. nigripes* adaptation to a flavonoid-rich plant *N. grossedentata* and inform the development of novel pest control strategies and the selection of resistant plant varieties.

## Simple Summary

In recent years, *Aoria nigripes* has inflicted significant damage on *Nekemias grossedentata*. However, the mechanism through which *A. nigripes* overcomes its host’s high flavonoid content to survive is still unclear, making it difficult to effectively prevent and control it. The present study is the first to investigate the effects of host plants with different flavonoid levels on the activities of three protective enzymes (SOD, POD, and CAT) and four detoxifying enzymes (CarE, AchE, GST, and CYP450) in *A. nigripes*, which provides important guidance for the further investigation of its adaptive mechanism and the development of pest control strategies. Overall, our findings indicate that the detoxifying enzymes GST and AchE and the protective enzyme POD play a major role in the response of *A. nigripes* to *N. grossedentata*, the “king of plant flavonoids”. Meanwhile, dihydromyricetin is an important flavonoid sed in *N. grossedentata* against insect injury stress. Additionally, the major flavonoids of *N. grossedentata* are enriched during digestion and metabolism in insects.

## 1. Introduction

Secondary plant metabolites exert deleterious effects on insect feeding behavior, growth, development, as well as reproduction and can be toxic to insects, thereby serving as a defense mechanism against insect herbivory [1]. Flavonoids, the category of the largest group of secondary plant products, are pivotal in plant defenses against pests [2,3]. Their concentration in fresh leaves is significantly enhanced when insects feed on Lingtou Danzong tea [4]. Furthermore, the content of these secondary metabolites can vary across different strains of the same plant [5,6,7,8], leading to differences in effects on insects [1]. In response to the detrimental impacts of secondary plant substances, insects adapt to their host plants via various strategies including avoidance of feeding on secondary plant substances and the induction of detoxification metabolism [1]. This involves the secretion of protective enzymes and detoxification enzymes, which is considered a critical adaptive response of insects to plants [9].

Superoxide dismutase (SOD), peroxidase (POD), and catalase (CAT) are essential protective enzymes in insects [10], while glutathione S-transferase (GST), carboxylesterase (CarE), acetylcholinesterase (AchE), and cytochrome P450 (CYP450) are the primary detoxification enzymes [9,11,12]. These enzymes play a significant role in the metabolic adaptation to secondary substances [13,14]. Upon entry into the insect body, secondary substances can stimulate the upregulation of metabolic enzyme gene expression and the production of protective enzymes to neutralize the free radicals produced by harmful substances, thereby safeguarding crucial tissues and organs [13,14]. Concurrently, the elevated activities of detoxification enzymes expedite the detoxification and metabolic processes of secondary substances, facilitating their degradation, utilization, or excretion from the body, thus mitigating or preventing the toxic effects of secondary substances [13,14]. Detoxification and protective enzyme activities in insects are contingent upon the type of host plant [9,15,16,17,18,19,20], and these activities differ among insects when they feed on various varieties of the same host plant species [9,21]. Investigating the effects of host plants on the activities of protective and detoxifying enzymes in insects can thus provide insights into the adaptive mechanisms of phytophagous insects to their hosts.

**Figure 1:**
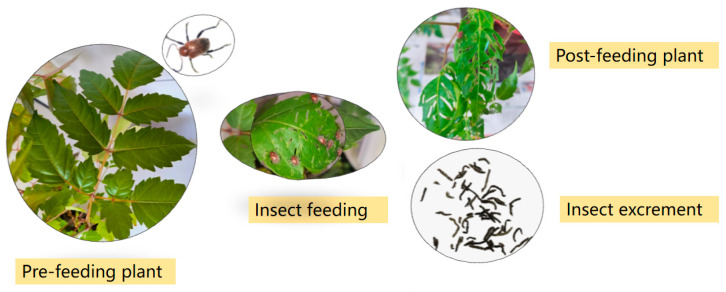
*Aoria nigripes* fetching *N. grossedentata*.

*Aoria nigripes* (Baly, 1860) is a species of Coleoptera, Chrysomelidae, and is widely distributed across China, Vietnam, Laos, Cambodia, Myanmar, Thailand, India, Indonesia, and other regions [22,23,24,25]. It is known to cause severe damage to Vitaceae plants, particularly grapes, wild grapes, *Parthenocissus himalayana*, and crystal grapes [23,24,26]. Adults primarily feed on the leaves, petioles, and shoots, as well as the adaxial leaf flesh of leaves and the epidermis of the branches and petioles, creating transparent feeding strips on the leaf blades [26,27]. This feeding weakens the plants and, in severe instances, can lead to plant death, resulting in significant economic losses (Figure 1). Studies on the occurrence and control of *A. nigripes* are limited, although recent research has focused on its biological characteristics and control methods [26,27,28,29]. However, no research has been conducted on the protective and detoxifying enzyme activities of this insect.

*Nekemias grossedentata* (Hand.-Mazz.) J. Wen & Z. L. Nie is a perennial woody vine plant in the family Vitaceae, recognized for its high flavonoid content, up to 45% in the dried leaves [30], and is esteemed as the “king of plant flavonoids” [29,31,32]. Its primary medicinal components include dihydromyricetin, myricitrin, and myricetin [33]. To date, research on the insect pests of *N. grossedentata* has been scarce. Our research team has documented damage to *N. grossedentata* caused by *A. nigripes* [29] and determined the complete mitochondrial genome of *A. nigripes* [28]. Adults damage the leaves, creating transparent feeding strips, while larvae target the roots and stems, leading to leaf yellowing or plant death, affecting its quality and yield [29]. As a medicine food homology plant, it holds significant economic value due to its antibacterial, antioxidant, antiviral, and anticancer properties [29,30,34]. The young leaves are primarily processed into products, and feeding by *A. nigripes* results in economic losses. However, on *N. grossedentata*, a plant renowned for its flavonoid content, *A. nigripes* can survive and reproduce without issue. This suggests that the insect has evolved a unique set of mechanisms for detoxifying flavonoids. Consequently, the present study is the first t examined the effects of *N. grossedentata* with varying flavonoid levels (high, middle, and low) on the activities of three protective enzymes (SOD, POD, and CAT) and four detoxifying enzymes (CarE, AchE, GST, and CYP450) in *A. nigripes*, along with changes in their principal flavonoids within the insect. This study also investigated the dynamic changes I the flavonoids in the leaves of the host plant before and after damage by *A. nigripes*. Based on correlation analysis, the physiological and biochemical mechanisms of the interaction between *N. grossedentata* and *A. nigripes* were elucidated. This research serves as a crucial foundation for further investigation into the molecular mechanisms of this insect and the developing novel prevention and control strategies.

## 2. Materials and Methods

### 2.1. Insects

The adults of *A. nigripes* were collected Pingba District, Anshun City, Guizhou Province, China (106°45′ E, 26°37′ N, 1244 m above sea level), and reared with crystal grape leaves in the laboratory at a temperature of 25 ± 1 °C, relative humidity of 65 ± 5%, and photoperiod of 14:10 h (L:D). *A. nigripes* adults of uniform size that were healthy and parasite-free were selected and starved for 3 h. At least 150 adults were placed in each cage, and three replicates were set up during the experiment, *N. grossedentata* cultivars with pots were placed in cages, and the leaves did not separate from the plant and the insects fed autonomously. Active *A. nigripes* adults were randomly collected from each group at 24 h, 48 h until 72 h; the elytra were removed, quick-frozen in liquid nitrogen, and then stored at −80 °C for use (the 0 h insect samples were collected after 3 h of starvation).

### 2.2. Host Plants

*N. grossedentata* were planted in large quantities in December 2022 at the germplasm resource nursery of Guizhou Normal University (106°63′ E, 26°38′ N) to ensure that the *N. grossedentata* from the cultivars were sufficient and had the same genetic background; they were watered regularly to strive for consistent growth conditions. Based on the content of dihydromyricetin, the most representative compound in *N. grossedentata*, the test plants were grouped as follows: low-content group (6.16% ± 0.66%), medium-content group (9.23% ± 1.19%), and high-content group (21.23% ± 1.23%), with the content approximately doubling in order from low to high. The leaves were collected 0, 24, 48 and 72 h after *A. nigripes* feeding, and the main leaf veins were removed and dried at a constant temperature of 60 °C until constant weight. The dried leaves were ground into powder and used to determine the contents of secondary metabolites.

### 2.3. Determination of Secondary Metabolite Contents of N. grossedentata

The total flavonoid contents in the leaves were determined by UV spectrophotometry [30], the standard solution was prepared with dihydromyricetin, and the optical density (OD) value was measured at the maximum absorption wavelength to obtain the standard curve. The HPLC method [30] was used to determine the contents of dihydromyricetin, myricitrin, and myricetin. HPLC analyses were carried out with an SHIMADZU LC-20ADXR series system (Shimadzu USA Manufacturing, Inc., Columbia, MD, USA) consisting of two LC-20AD pumps, an SPD-M20A diode array detector, and a SIL-20AC auto sampler. A CAPCELL PAK C18 column (4.6 × 250 mm, 5 µm) was adopted for the analyses. The mobile phase consisted of A (methanol) and B (0.1% phosphoric acid solution). The gradient mode was as follows: 0~20 min, 72% B; 20~21 min, 72~60% B; 21~30 min, 60% B; 30~31 min, 60~58% B; 31~45 min, 58% B; 45~46 min, 58~50% B; 46~50 min, 50% B; 50~51 min, 50~72% B; 51~60 min, 72% B. The flow rate was 1.0 mL/min. The detection wavelength was set at 292 (dihydromyricetin), 254 (myricitrin), and 375 (myricetin) nm. The column temperature was set at 40 °C, and injection volume was set at 10 µL.

### 2.4. Determination of Flavonoid Compounds in Insect Excreta

A10.00 mg sample was taken, weighed accurately, and put into a 10.0 mL stoppered tube; 5.0 mL of methanol was added; the tube was closed and sonicated for 40 min to obtain the sample solution, which was passed through 0.45 µm microporous filtration membrane; the filtrate was taken for HPLC analysis.

### 2.5. Measurement of Protective and Detoxifying Enzyme Activities in A. nigripes Adults

The treated adults were randomly sampled at 24, 48 and 72 h after *A. nigripes* feeding, and the number of samples in each experimental group at each time point was 50 individuals. For each enzyme activity assay, 6 adults were taken from each treatment, and 1.0 mL of the corresponding kit extract was added to the homogenate (*A. nigripes* with removed the elytra) in an ice bath. The homogenate was centrifuged for 10 min (12,000 r/min, 4 °C), GST for 15 min, and insect CYP450 at 8000 r/min; the supernatant was placed on ice for the assay.

CAT Assay Kit (product No.: RXWB0478-96), POD Assay Kit (product No.: RXWB0111-96), SOD Assay Kit (product No.: RXWB0482-96), GST Assay Kit (product No.: RXWB0099-96), CarE Assay Kit (product No: RXWB0181-96), AchE Detection Kit (product No.: RXWB0254-96) and Insect CYP450 ELISA Kit (product No.: RX1800390I) were purchased from Quanzhou Ruixin Biological Technology Co. Ltd., Quanzhou, China. The activity of each enzyme was determined according to the manufacturer’s instructions.

### 2.6. Statistical Analysis

The data were analyzed using Excel 2010 and SPSS 25.0 (IBM Corp., Armonk, NY, USA) software. Host secondary metabolite and enzyme activity data were analyzed using one-way analysis of variance (ANOVA), in which differences between means were compared at the *p* < 0.05 significance level using the Waller–Duncan multiple comparison test. Correlation between data were analyzed using Pearson linear correlation analysis. Correlation analyses between the data were carried out using Pearson’s linear correlation analysis to correlate the content of each compound at 0, 24, and 48 h of feeding on plants with the values of insect enzyme activities at 24, 48, and 72 h. The correlation analysis were performed using Pearson’s linear correlation method. Finally, GraphPad Prism 8.0.2 (GraphPad Software Inc., San Diego, CA, USA) software was used for graphing.

## 3. Results

### 3.1. Secondary Metabolites Contents in Host Plant

**Figure 2:**
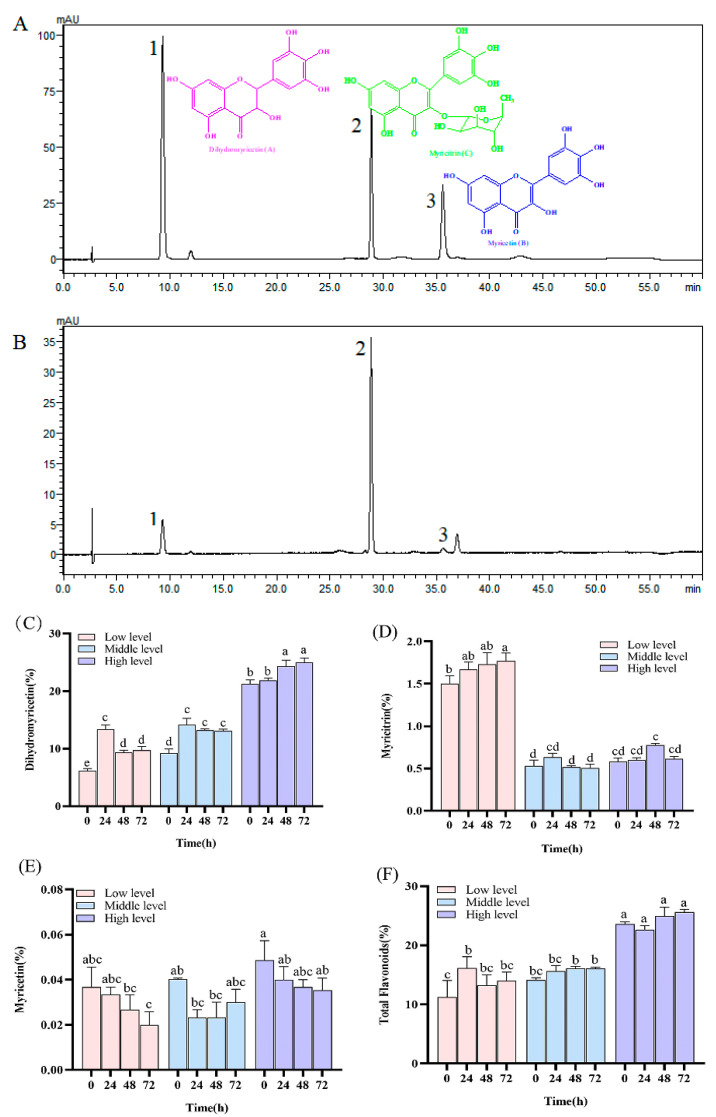
Changes in the content of major flavonoids and total flavonoids in *N. grossedentata*. (**A**) The mixed control, and (**B**) the test sample, where 1 is dihydromyricetin, 2 is myricitrin, 3 is myricetin. The dihydromyricetin content in *N. grossedentata* before insect feeding was used as the basis for grouping, and the content in the test plants was low (6.16% ± 0.66%), medium (9.23% ± 1.19%) and high (21.23% ± 1.23%). Note: The data in the figures are presented as mean ± SE, and different lowercase letters in (**C**–**F**) indicate a significant difference (*p* < 0.05).

Feeding *A. nigripes* on *N. grossedentata* exerted a deleterious effect on the plant’s growth, resulting in significant weakening. Subsequent analysis revealed variability in the secondary metabolite profiles within the *N. grossedentata* leaves from diverse strains that were characterized by distinct flavonoid levels. Notably, the primary flavonoids in *N. grossedentata* were dihydromyricetin, myricitrin, and myricetin, with elution times of 9.304 min, 28.866 min, and 35.561 min, respectively (Figure 2A,B). Among these, dihydromyricetin (Figure 2C) demonstrated statistically significant differences (*p* < 0.05) in concentration across the three strain types prior to feeding. The high-level strain (21.23% ± 1.23%) exhibited a content that was significantly greater (*p* < 0.05) than that of the low-level (6.16% ± 0.66%) and middle-level (9.23% ± 1.19%) strains, representing a 3.45-fold increase (*p* < 0.05) over the low-level strain. Myricitrin (Figure 2D) showed a significant increase (*p* < 0.05) in low-level leaves (1.50% ± 0.16%) compared to the middle-level (0.53% ± 0.12%) and high-level (0.58% ± 0.06%) strains before feeding. In contrast, myricetin (Figure 2E) exhibited no significant differences (*p* > 0.05) in content among the high-, middle-, and low-level strains before feeding, at 0.05% ± 0.01%, 0.04% ± 0.00%, and 0.04% ± 0.01%, respectively. The total flavonoid content (Figure 2F) was significantly elevated (*p* < 0.05) in the high-level strain (23.62% ± 0.59%) relative to the low-level (11.24% ± 4.89%) and middle-level (14.14% ± 0.66%) strains before feeding, representing a 2.10-fold increase over the low-level strain.

After 24, 48, and 72 h of feeding on *A. nigripes*, the changes in the contents of the primary and secondary metabolites in the host were different. Dihydromyricetin levels were found to rise with increasing feeding duration across all three strain types; compared to the baseline at 0 h, it reached maximum value at 24 h in the low-level and middle-level strains, while significant increases were observed at 48 h in the high-level strain (*p* < 0.05). The content of myricitrin exhibited fluctuations with feeding time, with the low-level strain demonstrating a continuous rise, reaching significance at 72 h (*p* < 0.05). the middle-level and high-level strains showed a pattern of increasing followed by decreasing, with no significant differences observed (*p* > 0.05). The myricetin content decreased uniformly across all strains after feeding, with the differences being non-significant (*p* > 0.05). Similarly, the total flavonoid content exhibited varying trends with the duration of feeding, with the low-level and middle-level strains showing an increase post-feeding, with the low-level strain exhibiting a significant increase at 24 h (*p* < 0.05). The high-level strain exhibited a decrease followed by an increase, with no significant differences observed (*p* > 0.05).

### 3.2. Three Protective and Four Detoxifying Enzyme Activities

Under the influence of high levels of flavonoids in the host, *A. nigripes* exhibited varying degrees of induction in the activities of four detoxifying enzymes (CYP450, CarE, GST, and AchE) and three protective enzymes (CAT, POD, and SOD) (Table 1). The mean values of enzyme activities at 24, 48 and 72 h were compared with the pre-feeding enzyme activities; under low-content stress, the sequence of induction in the detoxification enzyme activities was GST (1.66-fold) > CarE (1.26-fold) > AchE (1.14-fold) > CYP450 (0.98-fold); for protective enzyme activities, the sequence was POD (2.31-fold) > CAT (1.24-fold) > SOD (1.12-fold). In the context of medium -content stress, the detoxification enzyme activity sequence was AchE (1.80-fold) > CarE (1.61-fold) > GST (1.53-fold) > CYP450 (1.15-fold); for protective enzyme activities, the order of increase was POD (3.09-fold) > CAT (1.49-fold) > SOD (1.08-fold). Under high -content stress, the detoxification enzyme activity sequence was AchE (2.06-fold) ≈ GST (2.06-fold) > CarE (1.93-fold) > CYP450 (1.19-fold); and, for protective enzyme activities, the order was POD (2.88-fold) > CAT (1.36-fold) > SOD (1.10-fold).

#### 3.2.1. Superoxide Dismutase Activity

Superoxide dismutase (SOD) activity exhibited variations after *A. nigripes* feeding on three *N. grossedentata* strains with distinct flavonoid levels (Table 1). At 48 h post-feeding, the enzyme activity in the insects sourced from the high-level strain (162.31 ± 2.72 U/g) group was significantly reduced (*p* < 0.05) compared to the low-level (189.68 ± 4.44 U/g) and middle-level (199.36 ± 13.99 U/g) strains. At 72 h, the activity (187.44 ± 6.39 U/g) was significantly elevated (*p* < 0.05) relative to the middle-level strain (154.80 ± 18.20 U/g). The SOD activity in the insects displayed variable changes with extended feeding duration. For the high-level strain, the enzyme activity decreased and then increased, with the 48 h activity differing significantly from both the 24 h and 72 h activities (*p* < 0.05). In the low-level strain, the activity increased and then decreased, and there was no significant difference among the 24 h, 48 h, and 72 h activities (*p* > 0.05). For the middle-level strain, the activity also increased and then decreased, with the 48 h activity differing significantly from the 24 h and 72 h activities (*p* < 0.05).

#### 3.2.2. Peroxidase Activity

The peroxidase (POD) activity exhibited marked variability following feeding on *N. grossedentata* from three plant strains characterized by distinct flavonoid levels (Table 1). After 24 h of Feeding, insects from the high-level strain group demonstrated a significantly higher POD activity (173.79 ± 0.46 U/g) compared to those from the low-level strain (117.47 ± 5.81 U/g) (*p* < 0.05). At 48 h, the POD activity in *A. nigripes* in the high-level strain (137.65 ± 7.48 U/g) group was also significantly greater than that in the low-level strain (109.05 ± 9.05 U/g) group, which was notably lower than the POD activity in insects from the middle-level strain (194.12 ± 16.19 U/g) (*p* < 0.05). By 72 h, the POD activity in the high-level strain (155.00 ± 6.67 U/g) was found to be significantly higher than in the middle-level strain (122.94 ± 2.00 U/g) (*p* < 0.05). As feeding duration was prolonged, POD activity in the high-level strain decreased and then increased, with significant differences noted between 24 h, 48 h, and 72 h (*p* < 0.05). The low-level strain activity decreased and then increased, with a significant difference between 72 h and both 24 h and 48 h (*p* < 0.05). The middle-level strain activity increased and then decreased, with a significant difference between 72 h and both 24 h and 48 h (*p* < 0.05).

#### 3.2.3. CAT Activity

The catalase (CAT) activity in *A. nigripes* was altered after consuming *N. grossedentata* from three strains with differing flavonoid levels (Table 1). The enzyme activity for the high-level strain was significantly higher (*p* < 0.05) than that of the low-level strain for 24 h and 48 h. The 48-h activity was significantly higher (*p* < 0.05) than that of the low level and middle level strains, which was also significantly lower (*p* < 0.05) than that of the middle level strain. At 72 h, the activity (284.07 ± 3.37 U/g) was significantly lower (*p* < 0.05) than that of the middle-level (331.52 ± 25.78 U/g) and low-level (318.82 ± 8.49 U/g) strains. CAT activities varied with feeding time, with the high-level strain activity continuing to decrease, with significant differences between 24 h, 48 h, and 72 h (*p* < 0.05). The low-level strain activity first decreased and then increased, with significant differences between 48 h and 24 h and 72 h (*p* < 0.05). The middle-level strain activity continued to decrease, with significant differences between 72 h and 24 h and 48 h (*p* < 0.05).

#### 3.2.4. CarE Activity

The Carboxylesterase (CarE) activity in *A. nigripes* was distinct after feeding on *N. grossedentata* from three strains with different flavonoid levels (Table 1). The enzyme activity for the high-level strain at 72 h (71.22 ± 4.33 U/g) was significantly higher (*p* < 0.05) than that of the low-level (20.80 ± 3.36 U/g) and middle-level (38.06 ± 6.31 U/g) strains. The CarE activity of the insects exhibited varying changes with feeding time. The high-level strain activity continued to rise, with significant differences between 72 h and 24 h and 48 h (*p* < 0.05). The low-level strain activity rose and then declined, with significant differences between 24 h, 48 h, and 72 h (*p* < 0.05). The middle-level strain activity continued to fall, with significant differences between 24 h and 48 h and 72 h (*p* < 0.05).

#### 3.2.5. AchE Activity

The acetylcholinesterase (AchE) activity in *A. nigripes* varied following feeding on *N. grossedentata* from three strains with distinct flavonoid levels (Table 1). The enzyme activities at 24 h, 48 h and 72 h for the high-level strain were significantly higher than those of the low-level strain, which were also significantly higher (*p* < 0.05) than those of the middle-level strain at 72 h. The AchE activity of the insects changed with the duration of feeding. The high-level strain activity continued to increase, with significant differences between 72 h and 24 h and 48 h (*p* < 0.05). The low-level strain activity continued to decrease, with significant differences between 24 h, 48 h, and 72 h (*p* < 0.05). The middle-level strain activity first increased and then decreased, with significant differences between 24 h, 48 h and 72 h (*p* < 0.05).

#### 3.2.6. GST Activity

Glutathione S-transferase (GST) activity in *A. nigripes* displayed variability after feeding on three *N. grossedentata* strains with varying flavonoid levels (Table 1). The 24 h and 72 h enzyme activities for the high-level strain were significantly higher than those for the low-level and middle-level strains, and the high-level strain activity was also significantly higher (*p* < 0.05) than that of the middle-level strain at 48 h. The GST activity varied with feeding time, with the high -level strain activity decreasing and then increasing, with significant differences between 72 h and 48 h (*p* < 0.05). The low-level strain activity increased and then decreased, with significant differences between 72 h and 48 h (*p* < 0.05). The middle-level strain activity decreased and then increased.

#### 3.2.7. CYP450 Activity

Cytochrome P450 (CYP450) enzyme activity in *A. nigripes* was different after feeding on *N. grossedentata* from three strains with distinct flavonoid levels (Table 1). The enzyme activity for the high-level strain was significantly higher (*p* < 0.05) than that of the low- level and middle-level strains at 24 h, 48 h, and 72 h. The activity was significantly higher (*p* < 0.05) at 48 h than that of the middle-level strain, but significantly lower (*p* < 0.05) at 24 h than that of the middle-level strain. The CYP450 activity of the insects changed with feeding time, with the high-level strain activity continuing to increase, with significant differences between 48 h, 72 h, and 24 h (*p* < 0.05). The low-level strain activity continued to increase, with significant differences between 24 h, 48 h and 72 h (*p* < 0.05). The middle-level strain activity first decreased and then increased, with significant differences between 24 h, 48 h and 72 h (*p* < 0.05).

### 3.3. Relationship Between A. nigripes Enzyme Activity and Secondary Metabolite Content in Host Plant Leaves

Pearson’s linear correlation coefficient analysis (Table 2) revealed highly significant positive correlations between the activities of acetylcholinesterase (AchE), carboxylesterase (CarE), glutathione S-transferase (GST), and cytochrome P450 (CYP450) enzymes, as well as the contents of total flavonoids and dihydromyricetin. Conversely, these enzymes and activities exhibited a negative correlation with myricitrin content, with particularly strong negative correlations observed between AchE and CYP450 with myricitrin, and a significant negative correlation between CarE and myricitrin. AchE, CarE, and GST activities also correlated positively with myricetin content, whereas CYP450 activity was negatively correlated with myricetin. Peroxidase (POD) activity positively correlated with total flavonoids, dihydromyricetin, and myricetin content, and negatively and highly significantly correlated with myricitrin content. Superoxide Dismutase (SOD) activity positively correlated with total flavonoids, dihydromyricetin, myricitrin, and myricetin content. Catalase (CAT) activity negatively correlated with total flavonoids, dihydromyricetin, and myricitrin content, and positively correlated with myricetin content, with the negative correlation with myricitrin being highly significant.

### 3.4. Metabolic Accumulation of Major N. grossedentata Flavonoids in Insects

**Figure 3:**
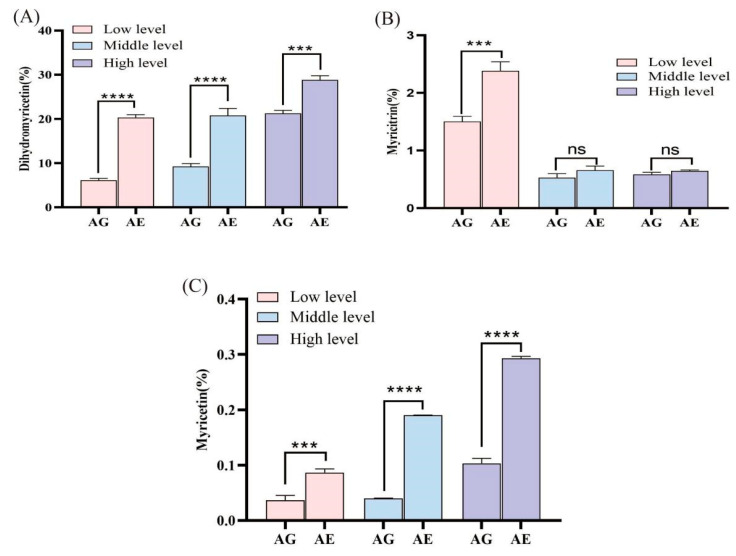
Metabolism of major flavonoids in *N. grossedentata* in *A. nigripes*. AG indicates *N. grossedentata* raw material, AE indicates excreta after insect feeding, *** indicates significant difference at the 0.001 level, **** indicates significant difference at the 0.0001 level, and ns indicates no significant difference. (**A**) Dihydromyricetin content, (**B**) Myricitrin content, (**C**) Myricetin content.

The study findings indicate that *A. nigripes* consumed *N. grossedentata* leaves with varying flavonoid concentrations; following in vivo digestion, the levels of dihydromyricetin, myricetin, and myricitrin were significantly elevated in the insect excreta compared to the raw material (dried leaves of *N. grossedentata*). The content of these flavonoids in the excreta was significantly greater (*p* < 0.001) across all groups except for myricitrin, the content of which was not statistically different (*p* > 0.05) between the medium- and high-content raw materials and the excreta. The alterations in dihydromyricetin content were observed as 0.36-fold, 1.25-fold and 2.31-fold increases in the high-, medium-, and low-flavonoid-content groups, respectively (Figure 3A). And, the contents of myricitrin were 0.11-fold, 0.25-fold and 0.59-fold higher in the high-, medium-, and low-flavonoid-content groups, respectively (Figure 3B). Similarly, the myricetin contents were 1.84-fold, 3.75-fold and 1.35-fold higher in the high-, medium-, and low-flavonoid-content groups, respectively (Figure 3C).

## 4. Discussion

This study investigated the temporal dynamics of the detoxifying and protective enzymes in *A. nigripes* upon feeding on *N. grossedentata* leaves from three strain types, compared the major flavonoid contents in the leaves of these strains, and analyzed their intercorrelations. These efforts were aimed at providing a theoretical framework for delving into the adaptive mechanisms of *A. nigripes* and establishing a basis for future pest management strategies. It is well established that secondary metabolites serve as the primary line of defense for plants against herbivorous insects [11]. These compounds can impact the activities of protective and detoxifying enzymes in insects [35,36]. Insects have evolved to counteract these various toxins by adjusting the expression of protective and detoxifying enzymes within their bodies [37,38,39]. Flavonoids, key components of plant secondary metabolites, are crucial in plant defenses against insects and are broadly distributed in plants [40]. Previous research has demonstrated that the activities of POD, SOD, and CAT enzymes are significantly elevated in insects like *Spodoptera frugiperda* when they consume leaves enriched with total phenols, tannins, and flavonoids [41]. Quercetin stress has been shown to induce a marked increase in P450 enzyme activity in the bodies and midguts of adult *Helicoverpa armigera* [42] as well as to stimulate GST activity in silkworms [43].

Plant secondary metabolites induce the production of superoxide radicals in insects, which, in turn, activate protective enzymes to shield the insects from oxidative damage [11,43,44]. When insects are exposed to exogenous compounds, SOD converts superoxide radicals (O^2−^) into hydrogen peroxide (H_2_O_2_); CAT then breaks down H_2_O_2_ into water (H_2_O) and oxygen (O_2_) when H_2_O_2_ levels are excessive, while POD acts when H_2_O_2_ concentrations are low [37,45,46]. This study revealed that the levels of total flavonoids, dihydromyricetin, and myricetin in *N. grossedentata* leaves from high-level and middle-level strains exceeded those from low-level strains, with the myricitrin content being the highest in the low-level strain. At 24 and 48 h post-feeding, the POD and CAT activities in *A. nigripes* consuming high-level and middle-level strain leaves were significantly higher than in those consuming low-level strain leaves, whereas the POD enzyme activities remained unchanged. Over time, the SOD activity exhibited minor fluctuations and positively correlated with several flavonoids; the CAT activity showed a declining trend and negatively correlated with total flavonoids, dihydromyricetin, as well as myricitrin and positively correlated with myricetin; the POD activity showed significant fluctuations and positively correlated with total flavonoids, dihydromyricetin, and myricitrin. Similar findings were observed in *Spodoptera frugiperda*, where the POD, SOD, and CAT activities were significantly increased in the F1 generation larvae fed on potato leaves with high total phenols, tannins, and flavonoids [11,41]. When comparing the mean enzyme activity values at 24, 48, and 72 h post-feeding with pre-feeding values, the order of the increases in protective enzyme activities under stress with low dihydromyricetin content in *N. grossedentata* was POD (2.31-fold) > CAT (1.24-fold) > SOD (1.12-fold); under medium-content stress, the order was POD (3.09-fold) > CAT (1.49-fold) > SOD (1.08-fold); and, under high-content stress, the order was POD (2.88-fold) > CAT (1.36-fold) > SOD (1.10-fold). Among the protective enzymes, POD was found to play a pivotal role in the detoxification and protection of *A. nigripes*, offering a foundation for further in-depth research.

Host plants can affect the detoxification enzyme activities of insects in addition to their protective enzymes. GST, CarE, AchE, and CYP450 are important detoxification enzymes in insects [11,12], which play important roles in the decomposition of exogenous poisons and the maintenance of normal physiological metabolism [36,41,47,48]. The results of this study showed that the CarE enzyme activity of *A. nigripes* fed on the high-level strain was significantly higher than that of *A. nigripes* feeding on low-level and middle-level strain at 72 h; the AchE enzyme activity at 24 h, 48 h and 72 h of feeding was significantly higher than that of feeding on the low-level strain; the GST enzyme activity at 24 h and 72 h of feeding was significantly higher than that of those feeding on low-level and middle-level strains; the CYP450 enzyme activity at 24 h, 48 h and 72 h of feeding was significantly higher than that of those feeding on the low-level strain. And, all four detoxification enzyme activities were highly significant positively correlated with the total flavonoid and dihydromyricetin contents as well as negatively correlated with the myricitrin content. These results are similar to previous studies, in which GST and CarE activities in the adults of *Monolepta hieroglyphica* increased dramatically after feeding on two host plants, *Lycopersicon esculentum* and *Artemisia selengensis* [49]. *Loxostege sticticalis* larvae showed a rapid increase in detoxification enzyme activity with feeding on unsuitable host plants as feeding time prolonged [19]. Feeding on both potato and tobacco leaves resulted in varying degrees of elevation of GST, CarE, MFO and CYP450 activities in *Spodoptera frugiperda* larvae [41]. These findings indicate that different insect species may have developed their own adaptation mechanisms to their host plants and that different host plants contain different types of secondary metabolites, which lead to differences in enzyme activities [9,50]. The mean values of enzyme activities at 24, 48 and 72 h were compared with pre-feeding enzyme activities; the order of increase in detoxification enzyme activities under *N. grossedentata* stress with a low content of dihydromyricetin was GST (1.66-fold) > CarE (1.26-fold) > AchE (1.14-fold) > CYP450 (0.98-fold); under medium-content stress, the order of increase in detoxification enzyme activities was AchE (1.80-fold) > CarE (1.61-fold) > GST (1.53-fold) > CYP450 (1.15-fold); the order of increase in detoxification enzyme activity under high-content stress was AchE (2.06-fold) ≈ GST (2.06-fold) > CarE (1.93-fold) > CYP450 (1.19-fold.; Among the four detoxification enzymes, GST and AchE play an important role in detoxification of harmful substances in *A. nigripes*, which is of great significance for the subsequent investigation of its detoxification mechanism.

It has been shown that one of the important defense strategies of plants against insects is changing the contents of secondary metabolites [40,51]; among them, flavonoids are one of the largest plant secondary products [52], which are widely found in plants and play an important role in plant defense against pests [40,53]. In the present study, the leaves of three *N. grossedentata* strains were fed to *A. nigripes*, which showed an increasing trend in dihydromyricetin, myricitrin, and total flavonoid contents after feeding, *N. grossedentata* showed different changes for each flavonoid component of the host after insect injury stress, suggesting that they play different roles in defending against insect stress. The content of dihydromyricetin showed the most obvious upward trend during the insect injury process, which was hypothesized to play an important role in the defense of the host against insect injury. Previous studies showed a significant increase in flavonoid content when fresh leaves of Lingtou Danzong tea were fed to *Basilepta melanopus* [4]. Metabolomics data of *Camellia sinensis* after feeding on *Colaspoides femoralis* showed significant enrichment in flavonoids and phenolic acids [3]. However, the impact of the dihydromyricetin content on *A. nigripes* is still unclear, and the molecular mechanisms of the *N. grossedentata* response to insect pests need to be further explored.

## 5. Conclusions

*A. nigripes* exhibited the ability to modulate the activities of protective and detoxification enzymes, thereby enhancing its adaptation to the secondary metabolites and toxic substances of *N. grossedentata,* particularly when ingesting leaves with varied flavonoid levels. This study demonstrated that the detoxifying enzymes glutathione S-transferase (GST) and acetylcholinesterase (AchE), as well as the protective enzyme peroxidase (POD), were particularly efficacious in mitigating the flavonoid-induced stress in *A. nigripes.* Concurrently, the levels of the primary flavonoids dihydromyricetin and myricitrin, which are prominent in *N. grossedentata*, increased following *A. nigripes* consumption, with a trend that closely mirrored that of dihydromyricetin for total flavonoids. This suggests a role of these flavonoids in the host’s defense against insect infestation. This research constitutes the first exploration of the interactions between *A. nigripes* and *N. grossedentata*, offering a foundational understanding of their adaptive mechanisms and laying the groundwork for future investigations into pest control strategies.

## Figures and Tables

**Table 1:** Effect of *N. grossedentata* with different flavonoids level on the enzymatic activity of *A. nigripes*.

Enzyme	t/h	Host Plant		
		Low Level	Middle Level	High Level
Acetylcholinesterase (U/g), AchE	0	89.76 ± 3.29 ^e^	89.76 ± 3.29 ^e^	89.76 ± 3.29 ^e^
	24	135.98 ± 2.53 ^d^	160.55 ± 3.32 ^c^	176.06 ± 3.38 ^b^
	48	102.17 ± 4.97 ^e^	182.32 ± 22.22 ^b^	178.81 ± 2.21 ^b^
	72	67.96 ± 1.38 ^f^	141.73 ± 1.56 ^d^	200.28 ± 1.83 ^a^
Carboxylesterase (U/g), CarE	0	27.46 ± 1.89 ^gh^	27.46 ± 1.89 ^gh^	27.46 ± 1.89 ^gh^
	24	33.21 ± 2.28 ^fg^	55.20 ± 4.58 ^b^	40.83 ± 2.15 ^de^
	48	49.56 ± 4.14 ^bc^	39.42 ± 2.80 ^ef^	47.16 ± 3.38 ^cd^
	72	20.80 ± 3.36 ^h^	38.06 ± 6.31 ^ef^	71.22 ± 4.33 ^a^
Glutathione S-transferase (U/g), GST	0	722.69 ± 79.50 ^f^	722.69 ± 79.50 ^f^	722.69 ± 79.50 ^f^
	24	1238.20 ± 3.51 ^cd^	1119.04 ± 3.91 ^cde^	1475.75 ± 48.71 ^ab^
	48	1313.40 ± 132.93 ^bc^	1013.06 ± 92.68 ^e^	1307.39 ± 146.88 ^bc^
	72	1043.91 ± 101.74 ^de^	1183.89 ± 176.17 ^cde^	1676.28 ± 141.90 ^a^
Cytochrome P450 (ng/g), CYP450	0	14.95 ± 0.88 ^d^	14.95 ± 0.88 ^d^	14.95 ± 0.88 ^d^
	24	12.47 ± 0.45 ^e^	16.96 ± 0.81 ^b^	15.46 ± 0.54 ^cd^
	48	15.05 ± 0.56 ^cd^	15.70 ± 1.04 ^bcd^	18.55 ± 0.21 ^a^
	72	16.45 ± 1.04 ^bc^	19.04 ± 0.13 ^a^	19.38 ± 0.61 ^a^
Peroxidase (U/g), POD	0	53.92 ± 8.61 ^g^	53.92 ± 8.61 ^g^	53.92 ± 8.61 ^g^
	24	117.47 ± 5.81 ^f^	182.14 ± 4.34 ^ab^	173.79 ± 0.46 ^b^
	48	109.05 ± 9.05 ^f^	194.12 ± 16.19 ^a^	137.65 ± 7.48 ^de^
	72	147.55 ± 6.48 ^cd^	122.94 ± 2.00 ^ef^	155.00 ± 6.67 ^c^
Superoxide Dismutase (U/g), SOD	0	163.03 ± 15.41 ^c^	163.03 ± 15.41 ^c^	163.03 ± 15.41 ^c^
	24	187.58 ± 7.45 ^ab^	172.05 ± 7.69 ^bc^	188.42 ± 5.41 ^ab^
	48	189.68 ± 4.44 ^ab^	199.36 ± 13.99 ^a^	162.31 ± 2.72 ^c^
	72	170.28 ± 12.73 ^bc^	154.80 ± 18.20 ^c^	187.44 ± 6.39 ^ab^
Catalase (U/g), CAT	0	253.18 ± 15.37 ^c^	253.18 ± 15.37 ^c^	253.18 ± 15.37 ^c^
	24	349.05 ± 15.7 ^b^	404.33 ± 18.86 ^a^	418.49 ± 17.96 ^a^
	48	274.96 ± 18.55 ^c^	396.13 ± 15.45 ^a^	327.70 ± 0.92 ^b^
	72	318.82 ± 8.49 ^b^	331.52 ± 25.78 ^b^	284.07 ± 3.37 ^c^

Note: Data in the table are mean ± SD, different lowercase letters indicate significant differences (*p* < 0.05), seven enzyme activities were analyzed separately for differences (data included enzyme activities at 0, 24, 48, and 72 h under the influence of three flavonoid levels of *N. grossedentata*), and enzyme activity at 0 h was pre-feeding.

**Table 2:** Pearson’s linear correlation coefficient between the activities of enzymes in *A. nigripes* and the content of secondary metabolites in plant leaves.

Enzyme	Secondary Metabolites			
	Total Flavonoids	Dihydromyricetin	Myricitrin	Myricetin
Acetylcholinesterase (U/g), AchE	0.583 **	0.633 **	−0.797 **	0.251
Carboxylesterase (U/g), CarE	0.563 **	0.561 **	−0.407 *	0.201
Glutathione S-transferase (U/g), GST	0.722 **	0.689 **	−0.143	0.370
Cytochrome P450 (ng/g), CYP450	0.525 **	0.579 **	−0.526 **	−0.047
Peroxidase (U/g), POD	0.178	0.208	−0.527 **	0.121
Superoxide dismutase (U/g), SOD	0.060	0.051	0.189	0.027
Catalase (U/g), CAT	−0.033	−0.123	−0.496 **	0.183

Note: The values in the table are Pearson’s correlation coefficient (r), * indicates correlation was significant at the 0.05 level (two-tailed), ** indicates correlation was significant at the 0.01 level (two-tailed).

## Data Availability

The raw data supporting the conclusions of this article will be made available by the authors on request.
